# Phosphate, calcium, and vitamin D signaling, transport, and metabolism in the endometria of cyclic ewes

**DOI:** 10.1186/s40104-022-00803-2

**Published:** 2023-01-12

**Authors:** Claire Stenhouse, Makenzie G. Newton, Katherine M. Halloran, Robyn M. Moses, Nirvay Sah, Larry J. Suva, Fuller W. Bazer

**Affiliations:** 1 Departments of Animal Science, Texas A&M University, Kleberg Center, TX 77843-2471 College Station, USA; 2 Veterinary Physiology and Pharmacology, Texas A&M University, TX 77843 College Station, USA

**Keywords:** Calcium, Endometrium, Ovine, Phosphate, Vitamin D

## Abstract

**Background:**

Recent evidence suggests important roles for progesterone (P4) and interferon tau in the regulation of calcium, phosphate, and vitamin D signaling in the uteri of pregnant sheep. However, the effects of P4 and estradiol (E2), with respect to the expression of their receptors PGR and ESR1, respectively, in uterine epithelia on mineral signaling during the estrous cycle has not been investigated. Estrous cycles of mature Suffolk ewes were synchronized, prostaglandin F2α was administered, and ewes were observed for estrus (designated as Day 0) in the presence of vasectomized rams. On Days 1, 9, or 14 of the estrous cycle, hysterectomies were performed.

**Results:**

25-hydroxyvitamin D was more abundant in plasma from ewes on Day 14 than Day 1 (*P* < 0.05). Expression of fibroblast growth factor receptor 2 (*FGFR2)*, a disintegrin and metalloprotease 17 (*ADAM17*), and parathyroid hormone-related protein (*PTHrP)* mRNAs was greater in endometria on Day 9 compared to Days 1 and 14 (*P* < 0.01). Similarly, expression of transient receptor potential cation channel subfamily V member 6 (*TRPV6*) mRNA was greater in endometria on Day 9 than Day 1 (*P* < 0.05). ATPase plasma membrane Ca^2^^+^ transporting 4 (*ATP2B4*) and S100 calcium binding protein G (*S100G)* mRNA expression was greater in endometria on Day 14 than on Days 1 and 9 (*P* < 0.01). In contrast, endometrial expression of vitamin D receptor (*VDR*) mRNA was lower on Days 9 and 14 than Day 1 (*P* < 0.01). Expression of klotho (*KL*) (*P* < 0.05) and cytochrome P450 family 24 subfamily A member 1 (*CYP24*) (*P* < 0.01) mRNAs was lower on Day 14 than Days 1 and 9. ADAM17, FGF23, CYP2R1, CYP27B1, KL, and VDR proteins immunolocalized to the uterine myometrium, blood vessels, and uterine luminal (LE), superficial glandular (sGE), and glandular (GE) epithelia. S100A9 protein was weakly expressed in the uterine myometrium, LE, sGE, and GE. Immunoreactivity of CYP2R1 and KL proteins in uterine LE and sGE was less on Day 1 than on Days 9 and 14. In contrast, S100G protein was expressed exclusively by GE, and immunoreactive S100G protein was less on Day 9. S100A12 protein localized to stromal cells of the uterine stratum spongiosum and blood vessels, but not by uterine epithelial cells.

**Conclusion:**

Collectively, these results implicate E2, P4, and PGR in the regulation of phosphate, calcium, and vitamin D signaling in cyclic ewes.

**Supplementary Information:**

The online version contains supplementary material available at 10.1186/s40104-022-00803-2.

## Introduction

Secretions from the uterine epithelia are critical for growth and development of the conceptus (embryo/fetus and associated placental membranes) prior to the establishment of a functional placenta [1]. These nutrient-rich uterine secretions (known as histotroph) contain important molecules for the regulation of cellular functions including water, amino acids, hexose sugars, ions, growth factors, hormones, vitamins, and minerals [2]. Importantly, the composition of these uterine secretions varies across the estrous cycle, and between cyclic and pregnant animals, indicating regulation of histotrophic secretions by both paracrine and endocrine signaling.

It is well-established that phosphate, calcium, and vitamin D are critical regulators of renal and skeletal development and function [3]. Vitamin D, klotho (KL), parathyroid hormone (PTH), and the sex steroids [3–5] are important hormonal regulators of calcium and phosphate transport, absorption, and homeostasis in postnatal life. Further, the regulation of mineral metabolism postnatally relies upon the hormonal regulation of the expression of regulatory molecules such as calcium-transporting ATPases, calcium-binding proteins, sodium dependent phosphate transporters, and the transient receptor potential vanilloid (TRPV) family members [6–12]. Additionally, many studies have provided evidence for non-classical functions of phosphate, calcium, and vitamin D in multiple tissue types, with important roles in the regulation of many physiological processes including cellular metabolism, proliferation, and protein synthesis [3, 7, 13–17]. Recent evidence suggests that many of the postnatal regulatory mechanisms for phosphate, calcium, and vitamin D signaling, metabolism, and transport are present at the ovine maternal-conceptus interface [3, 18–21]. We recently reported that sodium-dependent phosphate transporters [solute carrier family 20 member 1 (SLC20A1) and SLC20A2], calcium-binding proteins [S100 calcium-binding protein G (S100G), S100A9, and S100A12], calcium transporters [ATP2B3, ATP2B4, TRPV5, and TRPV6], KL signaling components [fibroblast growth factor receptor (FGFR)1–4, FGF7, FGF21, FGF23, KL, a disintegrin and metalloproteinase domain-containing protein 10 (ADAM10) and ADAM17], and regulators of vitamin D signaling [cytochrome p450 family 2 subfamily R member 1 (CYP2R1), CYP24, CYP11A1, CYP27B1, and vitamin D receptor (VDR)] are expressed at the ovine maternal-conceptus interface throughout pregnancy [18, 19]. As such, these molecules have the potential to regulate the active transport of minerals from the maternal circulation into the fetal-placental circulation where they are essential for the regulation of fetal development [3, 21].

Progesterone (P4) is an essential regulator of uterine functions required for the establishment and maintenance of pregnancy in all mammalian species [22]. The ovine corpus luteum produces substantial quantities of P4 by Day 3 of the estrous cycle, with concentrations in maternal plasma of approximately 4 ng/mL by Day 7 [23]. Exposure of the ovine uterus to P4 for 8–10 d down-regulates expression of the progesterone receptor (PGR) in the uterine luminal (LE) and superficial glandular (sGE) epithelia, but does not affect expression of PGR in the deep uterine glandular epithelial cells (GE), stromal cells, or myometrium [24]. The down-regulation of PGR expression induces alterations in the endometrial expression of many nutrient transporters, making this event critical for the regulation of the secretion of histotroph into the uterine lumen [25]. In a cycling ewe, the corpus luteum begins to regress from approximately Days 14–15, and production of P4 declines sharply. This change is accompanied by increased secretion of estradiol (E2) by the maturing Graafian follicle which will be ovulated in the next estrous cycle. In the ovine uterus, E2 increases uterine estrogen receptor alpha (ESR1) and PGR expression whereas P4 inhibits ESR1 expression (reviewed by [26]). In cyclic ewes, regression of the corpus luteum allows the ewe to return to estrus, completing the 16–17-day estrous cycle. In cyclic ewes, ESR1 mRNA and protein expression in the endometrium is greatest on Day 1, significantly declining by Day 6, and then increasing between Days 11 and 15 [24].

Studies in both pigs and sheep indicate that calcium and phosphate increase in uterine flushings during the peri-implantation period of pregnancy, and are more abundant in uterine flushings from pregnant than cyclic animals during the mid-luteal phase of the estrous cycle [18, 19, 27, 28]. Further, temporal changes in the abundance of minerals in the uterine lumen during the peri-implantation period are accompanied with striking alterations in the expression of molecules with regulatory roles in phosphate, calcium, and vitamin D transport and signaling [18, 19]. Administration of P4 (the hormone of pregnancy) and/or interferon tau (IFNT, the pregnancy recognition signal in ruminants) to cyclic ewes results in alterations in circulating phosphate and 25-hydroxyvitamin D (25(OH)D; vitamin D metabolite) abundance [29]. This was accompanied by alterations in the endometrial expression of regulatory mRNAs and proteins affecting mineral transport and function [29], suggesting potential endocrine and/or paracrine regulation of systemic and local calcium, phosphate, and vitamin D signaling by the uterus. Thus, hormonal changes during the peri-implantation period of pregnancy appear to be critical for the local regulation of phosphate, calcium, and vitamin D signaling, transport and metabolism in the female reproductive tract. However, the effects of P4, with respect to expression of PGR in uterine epithelia (LE, sGE, GE), and E2 and ESR1 on mineral signaling in the ovine endometrium are not known.

This study aimed to examine the hormonal regulation of phosphate, calcium, and vitamin D signaling on Days 1, 9, and 14 of the estrous cycle, providing a platform for continued mechanistic investigation of mineral signaling, transport, and utilization deployed in the endometria of livestock species. Given the high rate of embryonic mortality in ruminants (20%–40%) with the majority of these losses occurring during the pre- and peri-implantation periods of pregnancy [30–32], it is imperative to improve our understanding of the mechanisms regulating histotroph secretion.

## Materials and methods

### Experimental animals and sample collection

Estrous cycles of mature Suffolk ewes (*n* = 13) were synchronized using a P4 intravaginal insert (CIDR, Zoetis, Parsippany, New Jersey, USA) for 12 d followed by an intramuscular injection of prostaglandin F2α (20 mg Lutalyse, Zoetis) upon CIDR removal. Following CIDR removal, ewes were observed for estrus (designated as Day 0) in the presence of a vasectomized ram. All ewes had exhibited a minimum of two estrous cycles of normal duration (16–18 d) prior to synchronization of estrus. Ewes were assigned randomly to be euthanized and hysterectomized after collecting blood via jugular venipuncture in an EDTA-coated vacutainer tube on either Day 1 (E2 dominant, PGR/ESR1 positive LE/sGE; *n* = 4), Day 9 (P4 dominant, PGR positive LE/sGE; *n* = 4), or Day 14 (P4 dominant, PGR negative LE/sGE; *n* = 5) of the estrous cycle. The uteri were flushed with 10 mL phosphate buffered saline (pH 7.2) and the total recovered volume of uterine flushings recorded; however, those values were not affected significantly by day of the estrous cycle. Cross sections of intact uteri were fixed overnight in 4% paraformaldehyde (Electron Microscopy Services, Hatfield, Pennsylvania, USA) and stored in 70% ethanol prior to embedding in paraffin wax. Endometrium was dissected from the myometrium of the uterus, frozen in liquid nitrogen, and stored at −80 °C. Plasma was collected following centrifugation of blood (8000 × *g* for 10 min at 4 °C) and stored at −20 °C until analyzed. Uterine flushings were centrifuged at 10,000 × *g* for 15 min at 4 °C, and the supernatant was stored at −20 °C until analyzed.

### Quantification of phosphate, calcium, and 25(OH)D in uterine flushings, plasma, and endometrial homogenates

Snap-frozen endometrial samples (300–500 mg of tissue) were homogenized in 1 mL of lysis buffer (60 mmol/L Tris-HCl (Sigma Aldrich, St. Louis, Missouri, USA), 1 mmol/L Na_3_VO_4_ (Fisher Scientific, Waltham, Massachusetts, USA), 10% glycerol (Fisher Scientific), 1% sodium dodecyl sulfate (BioRad, Hercules, California, USA), containing an EDTA-free protease inhibitor (Roche, Indianapolis, Indiana, USA). Homogenates were centrifuged at 14,000 × *g* for 15 min at 4 ^o^C, and the supernatant was removed and stored at −80 ^o^C until assayed. The concentrations of total proteins in the tissue homogenates were quantified spectrophotometrically (SynergyH1, BioTek, Shoreline, Washington, USA) using a protein assay dye reagent (BioRad; 500-0006) according to the manufacturer’s instructions.

The concentrations of phosphate in uterine flushings, plasma, and endometrial homogenates were quantified using a colorimetric assay (Abcam, Cambridge, Massachusetts, USA; ab65622), as described previously [18]. The absorbance of the plate was read on a spectrophotometric plate reader (SynergyH1, BioTek). Kit standards were used to generate a standard curve from 0 to 5 nmol/well and samples were diluted in double distilled water to ensure that concentrations were within the limits of the standard curve. The lower limit of detection of the assay was 0.1 nmol.

The concentrations of calcium in uterine flushings and endometrial homogenates were quantified using a colorimetric assay (Sigma Aldrich; MAK022), as described previously [19]. The absorbance of the plate was read on a spectrophotometric plate reader (SynergyH1, BioTek). Kit standards were used to generate a standard curve from 0 to 2 µg/well and samples were diluted in nuclease-free water to ensure that concentrations were within the limits of the standard curve.

The concentrations of 25(OH)D in plasma and endometrial homogenates were quantified by ELISA (Eagle Biosciences, Amherst, NH, USA; VID91-K01), as described previously [33]. Absorbance was read on a spectrophotometric plate reader (SynergyH1, BioTek) at 450 nm. The supplied kit standards were used to generate a standard curve from 0 to 150 ng/mL and samples were diluted in nuclease-free water to ensure that concentrations were within the detection limits of the standard curve.

Calcium and phosphate in uterine flushings were expressed as total calcium or total phosphate (volume of fluid × concentration). Calcium, phosphate, and 25(OH)D in endometrial homogenates was expressed relative to the concentration of protein in the sample.

### Analysis of candidate gene expression by qPCR

The relative expression of candidate genes with roles in calcium, phosphate, and vitamin D signaling, transport, and metabolism in endometria were quantified by qPCR as described previously [18, 19]. The candidate mRNAs investigated have roles that influence: phosphate (*SLC20A1, FGFR1*, *FGFR2*, *FGF23*, *KL*, *ADAM10* and *ADAM17*); calcium (*S100G*, *S100A9*, *ATP2B4*, parathyroid hormone-related protein [PTHrP], and *TRPV6*); and vitamin D (*CYP2R1*, *CYP24*, and *VDR*) signaling.

RNA was extracted from snap-frozen endometrial samples as described previously [29]. The RNA was quantified spectrophotometrically (NanoDrop ND-1000 Spectrophotometer), and all samples had a 260/280 value greater than 2. Complementary DNA (cDNA) was synthesized from 1 µg of RNA with SuperScript II reverse transcriptase and oligo (deoxythymidine) primers (Invitrogen, Carlsbad, CA, USA), as per the manufacturer’s instructions. Negative controls without reverse transcriptase were included to test for genomic contamination and all cDNA was stored at −20 ºC until required.

Quantitative polymerase chain reaction (qPCR) was performed using the ABI prism 7900HT system (Applied Biosystems, Foster City, CA, USA) with Power SYBR Green PCR Master Mix (Applied Biosystems), as per the manufacturer’s instructions to determine the levels of expression of mRNAs encoding for genes of interest. Primer sequences are provided in Additional file 1: Table S1. Primer efficiency and specificity were tested by generating a standard curve from pooled cDNA and by the inclusion of a dissociation curve to the RT reaction, respectively. Serial dilutions of pooled cDNA in nuclease-free water ranging from 1:2 to 1:256 were used as standards. All primer sets used amplified a single product and had an efficiency of between 95% and 105%. Each well contained 10% cDNA, 30% nuclease-free water, 10% primer, and 50% SYBR Green reaction mix in a 10-µL reaction volume. All reactions were performed at an annealing temperature of 60 ºC. For *FGF23*, lower expression was observed and 1 µL of cDNA was used in a pre-amplification step [34] using a Thermocycler (Eppendorf, Enfield, CT, USA). The stability of reference genes was assessed by geNORM V3.5 (Ghent University Hospital, Centre for Medical Genetics, Ghent, Belgium) in each tissue, with an M value of < 1.5. The reference genes tyrosine 3-monooxygenase/tryptophan 5-monooxygenase activation protein zeta (*YWHAZ*) and beta-2-microglobulin (*B2M*) were determined to have stable expression across the days of the estrous cycle according to evaluations using geNORM V3.5 (Ghent University Hospital, Centre for Medical Genetics). Additionally, effects of day of estrous cycle were determined using GenStat (Version 13.1; VSN International Ltd.) to ensure that there was no effect of day on reference gene expression. The abundances of the candidate mRNAs in the samples were quantified using the ΔΔCq method.

### Immunohistochemistry

Cell-specific immunohistochemical localization was performed for ADAM17, CYP2R1, CYP27B1, FGF23, KL, S100A9, S100A12, S100G, and VDR in endometria (Additional file 2: Table S2). Paraffin-embedded Sect. (5 μm) were deparaffinized in CitriSolv (Fisher Scientific) and rehydrated through a graded series of ethanol to double distilled water. Heat-induced epitope retrieval was performed in either sodium citrate buffer (pH 6.0) or in Tris-buffer (pH 9.0; Vector Laboratories, Burlinghame, California, USA). Endogenous peroxidase activity was blocked by incubation with 0.3% hydrogen peroxide (Sigma Aldrich) in methanol, and non-specific binding sites were blocked by incubation with normal horse serum (Vectastain Elite Universal ABC kit; Vector Laboratories) for 1 h at room temperature. Sections were incubated with a primary antibody (Additional file 2: Table S2) or with rabbit immunoglobulin G (rIgG) (Vector Laboratories) at the same concentration of total protein as a negative control. The slides were incubated overnight in a humidified chamber at 4 ^o^C, washed in PBS, and incubated for 1 h at 37 ^o^C in a humidified chamber with a biotinylated anti-rabbit IgG secondary antibody (Vectastain Elite ABC kit; Vector Laboratories) at 0.005 mg/mL in PBS containing 1.5% normal horse serum. Sections were incubated with Vectastain Elite ABC reagent (Vectastain Elite ABC kit; Vector Laboratories) for 30 min at 37 ^o^C in a humidified chamber. Slides were washed in 0.05 mol/L Tris-HCl and incubated with diaminobenzidine-tetra-hydrochloride hydrate (Sigma Aldrich) in 0.05 mol/L Tris-HCl containing hydrogen peroxide. Sections were counterstained with hematoxylin and dehydrated in a graded series of ethanol and CitriSolv (Fisher Scientific) before coverslips were affixed using Permount mounting medium (Fisher Scientific). Digital images of representative fields were recorded under brightfield illumination using a Nikon Eclipse microscope and NIS-Elements AR 4.30.02 64-bit Software (Nikon Instruments Inc, Melville, New York, USA).

### Statistical analysis

All statistical analyses were performed using GenStat 13.1. Mean values were calculated for each individual sample for each parameter investigated and the normality of the distribution of the data was assessed using the Anderson-Darling test. If a *P* value of < 0.05 was obtained, the data were not considered to have a normal distribution. Transformations were carried out if necessary to achieve a Gaussian distribution. Outliers identified by a ROUT outlier test were excluded. ANOVA with a Tukey post-hoc analysis was performed for analysis of temporal changes across the estrous cycle. The results were considered significant at *P* < 0.05, trending towards significant at 0.05 < *P* < 0.1 and not significant at *P* > 0.1.

## Results

### Phosphate, calcium, and 25(OH)D in uterine flushings, plasma, and endometrial homogenates

**Fig. 1:**
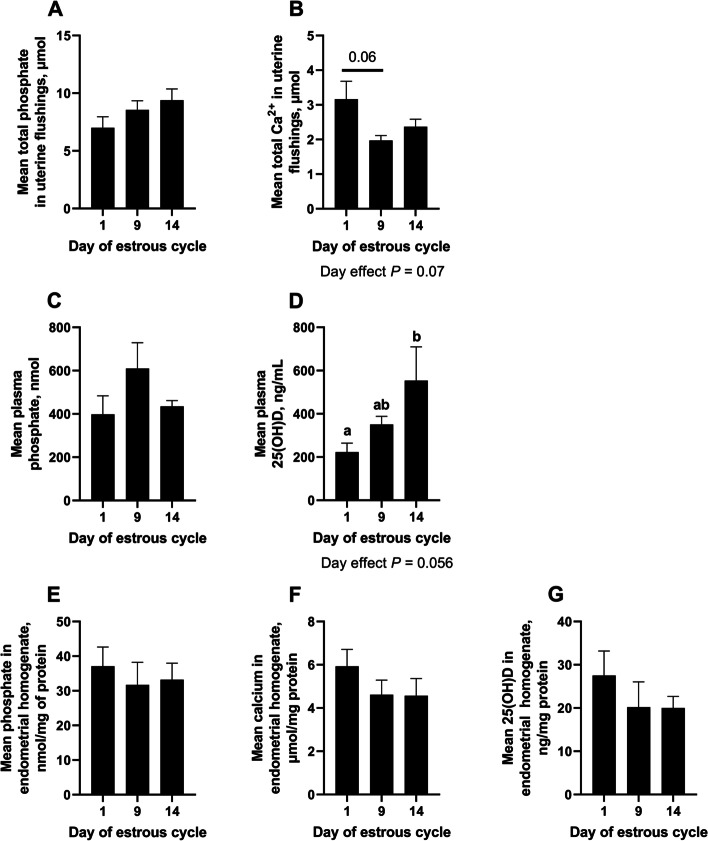
Quantification of phosphate, calcium, and 25(OH)D in uterine flushings, endometrial homogenates, and plasma. Phosphate was quantified in uterine flushings (**A**), plasma (**C**), and endometrial homogenates (**E**) using a colorimetric assay. Similarly, calcium was quantified in uterine flushings (**B**), and endometrial homogenates (**F**) using a colorimetric assay. 25(OH)D was quantified in plasma (**D**) and endometrial homogenates (**G**) by ELISA. Error bars represent SEM. Different letters indicate that means differ from one another (*P* < 0.05). *n* = 4–5 per day

Total calcium in uterine flushings tended to be less on Day 9 compared to Day 1 of the estrous cycle (*P* = 0.06; Day effect *P* = 0.07, Fig. 1B). Plasma 25(OH)D concentration increased between Day 1 and 14 of the estrous cycle (*P* < 0.05, Day effect *P* = 0.056; Fig. 1D). In contrast, total phosphate in uterine flushings, plasma, and endometrial homogenates was unaffected by day of the estrous cycle (Fig. 1). Similarly, calcium and 25(OH)D in endometrial homogenates were not affected by day of the estrous cycle (Fig. 1).

### Effects of day of the estrous cycle on endometrial expression of mRNAs involved in mineral metabolism and transport

#### Phosphate

**Fig. 2:**
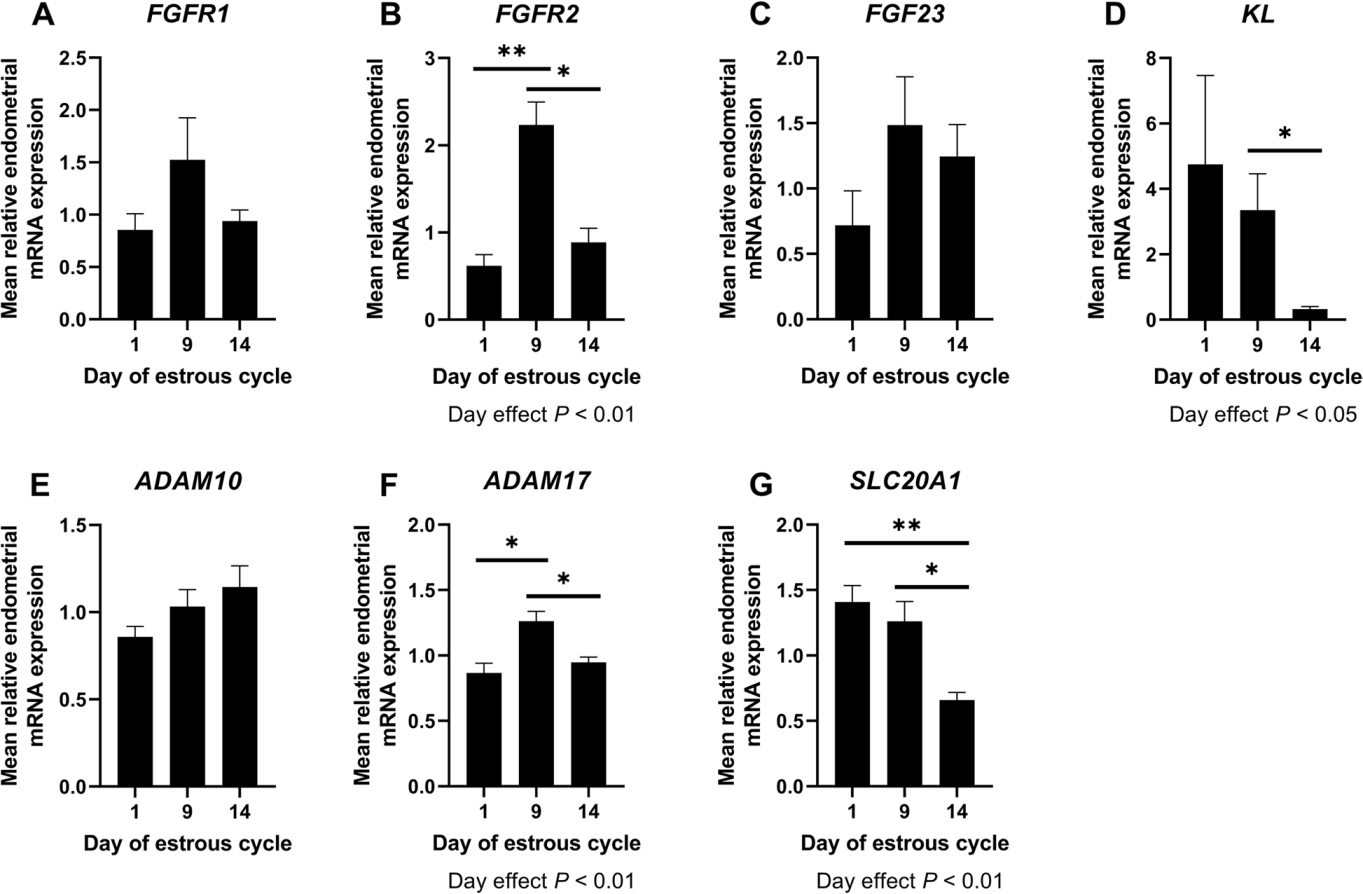
Quantification of mRNAs with roles in phosphate signaling and transport. The mRNA transcripts for *FGFR1* (**A**), *FGFR2* (**B**), *FGF23* (**C**), *KL* (**D**), *ADAM10* (**E**), *ADAM17* (**F**), and *SLC20A1* (**G**) were quantified in endometria using qPCR. Different letters indicate that group means differ (*P* < 0.05). Error bars represent SEM. *n* = 4–5 per day. *P < 0.05. **P < 0.01

The expression of *FGFR2* and *ADAM17* mRNAs (important components of Klotho-FGF signaling cascade) was greater on Day 9 of the estrous cycle than on Days 1 and 14 (Day Effect *P* < 0.01, Fig. 2B and F). Similarly, the expression of *KL* decreased between Day 9 and Day 14 of the cycle (Day effect *P* < 0.05, Fig. 2D). Expression of *SLC20A1* mRNA, a sodium dependent phosphate transporter, was lower on Day 14 of the estrous cycle compared to Days 1 and 9 (Day effect *P* < 0.01, Fig. 2G). Day of the estrous cycle did not affect endometrial expression of *FGFR1, FGF23*, or *ADAM10* mRNAs (*P* > 0.10, Fig. 2A, C, and E).

#### Calcium

**Fig. 3:**
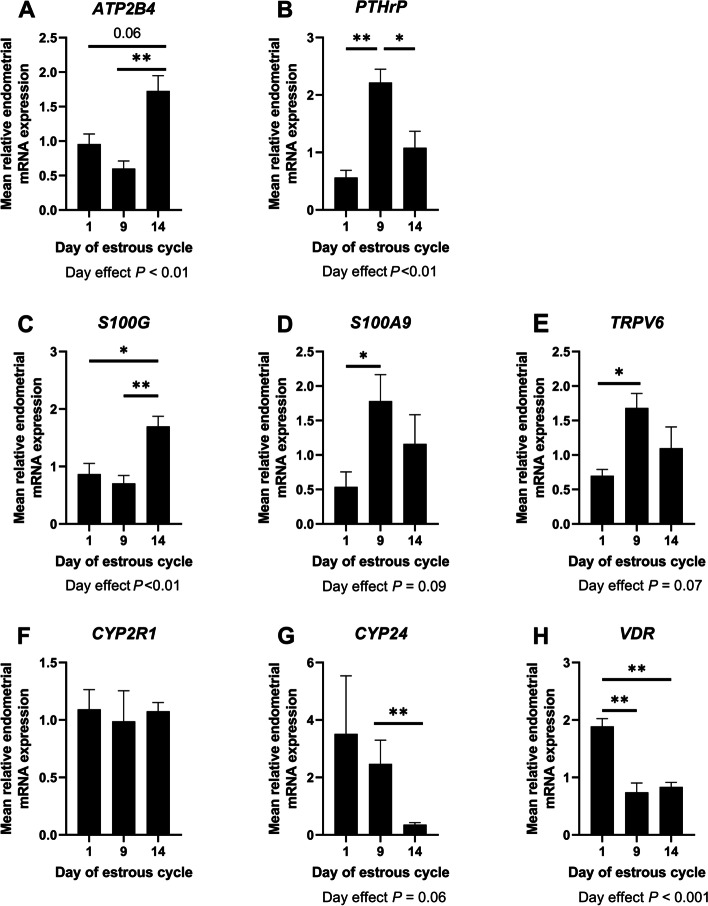
Quantification of mRNAs with roles in calcium and vitamin D signaling, transport, and metabolism. The mRNA transcripts for *ATP2B4* (**A**), *PTHrP* (**B**), *S100G* (**C**), *S100A9* (**D**), *TRPV6* (**E**), *CYP2R1* (**F**), *CYP24* (**G**), and *VDR* (**H**) were quantified in endometria using qPCR. Different letters indicate that group means differ (*P* < 0.05). Error bars represent SEM. *n* = 4–5 per day. *P < 0.05. **P < 0.01

The expression of *ATP2B4*, a plasma membrane calcium ATPase, and the calcium binding protein *S100G* was greater in endometria on Day 14 compared to Days 1 and 9 of the estrous cycle (Day effect *P* < 0.01, Fig. 3A and C). Expression of *PTHrP* mRNA expression was greater in endometria on Day 9 of the estrous cycle compared to Days 1 and 14 (Day effect *P* < 0.01, Fig. 3B). Expression of *S100A9* (*P* < 0.05, Day effect *P* = 0.09; Fig. 3D) and *TRPV6* (*P* < 0.05, Day effect *P* = 0.07; Fig. 3E) mRNAs was greater on Day 9 compared to Day 1 of the estrous cycle.

#### Vitamin D

Endometria on Day 14 of the estrous cycle had lower expression of the mRNA encoding 24-hydroxylase (*CYP24*) (important for the catabolism of vitamin D), compared to Day 9 of the estrous cycle (*P* < 0.01, Day effect *P* = 0.06, Fig. 3G). Similarly, expression of the vitamin D receptor (VDR) was lower in endometria on Days 9 and 14 of the estrous cycle compared to Day 1 (Day effect *P* < 0.001, Fig. 3H). Day of the estrous cycle did not affect endometrial expression of the mRNA encoding 25-hydroxylase, *CYP2R1* (Fig. 3F; *P* > 0.10).

### Immunolocalization of FGF23, KL, ADAM17, S100G, S100A9, S100A12, CYP2R1, CYP27B1, and VDR proteins in ovine endometria on Days 1, 9, and 14 of the estrous cycle

**Fig. 4:**
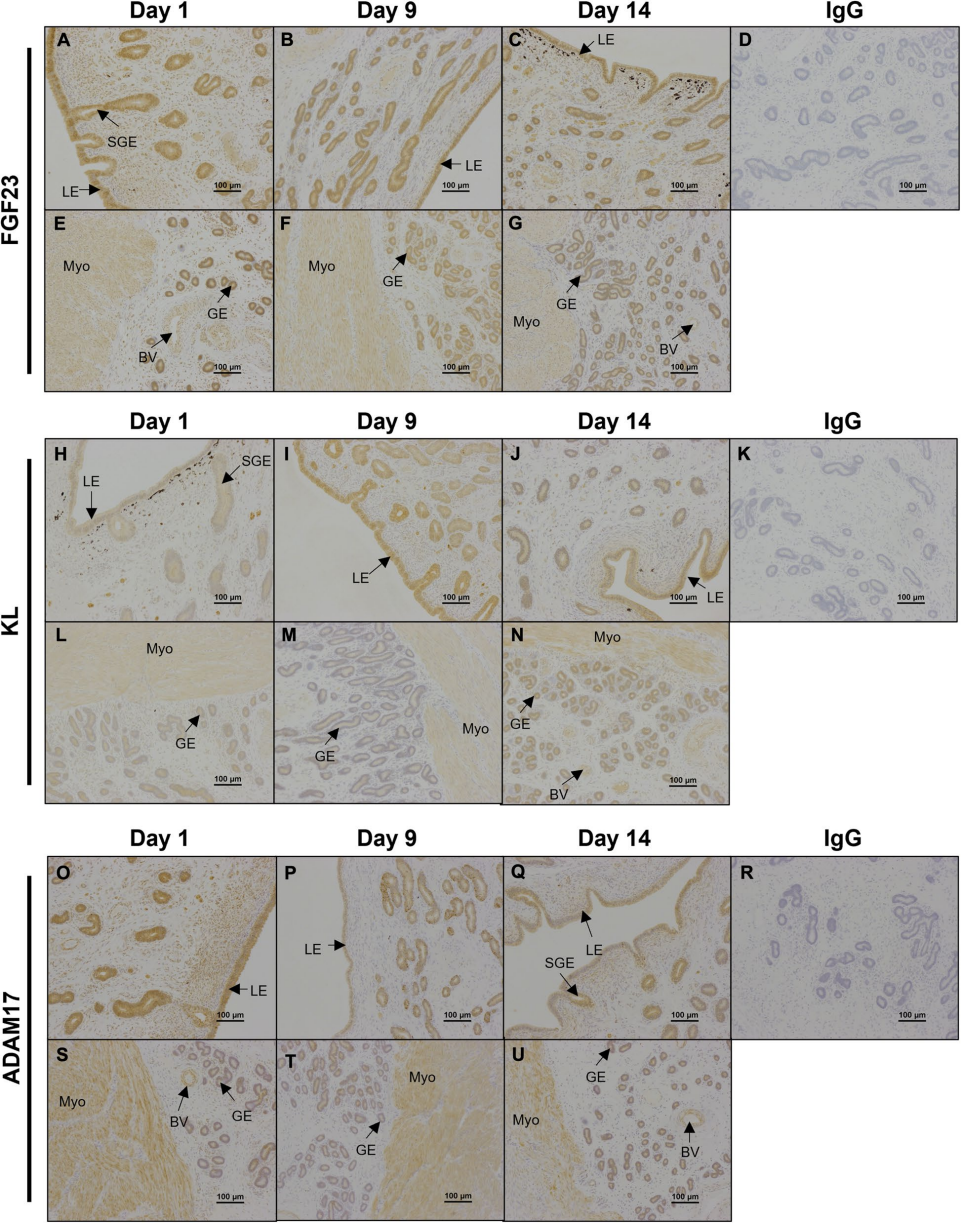
Representative immunolocalization of FGF23, KL, and ADAM17 proteins in ovine endometria. Representative images of immunohistochemical localization of FGF23 (**A**-**G**), KL (**H**-**N**), and ADAM17 (**O**-**U**) proteins in ovine endometria on Days 1, 9, and 14 of the estrous cycle. Rabbit IgG (rIgG) controls were included at equivalent protein concentrations to the antibody of interest as a negative control (D, K, R). BV = blood vessels, LE = luminal epithelium, GE = glandular epithelium, sGE = superficial glandular epithelium, Myo = myometrium. Scale bars represent 100 μm

To examine protein localization, immunohistochemistry was performed on uterine sections. FGF23 and ADAM17 proteins immunolocalized to the uterine LE, sGE, GE, blood vessels, myometrium, and some stromal cells (Fig. 4). Interestingly, while immunoreactive ADAM17 was present in the uterine stroma on Days 1 and 14 of the estrous cycle, particularly in the stratum compactum stroma, ADAM17 protein was not detectable there on Day 9. KL protein immunolocalized to the uterine LE, sGE, GE, myometrium, and blood vessels (Fig. 4). Immunoreactive KL protein was more abundant in uterine LE on Day 9 compared to Day 1 of the estrous cycle (Fig. 4H and I).

**Fig. 5:**
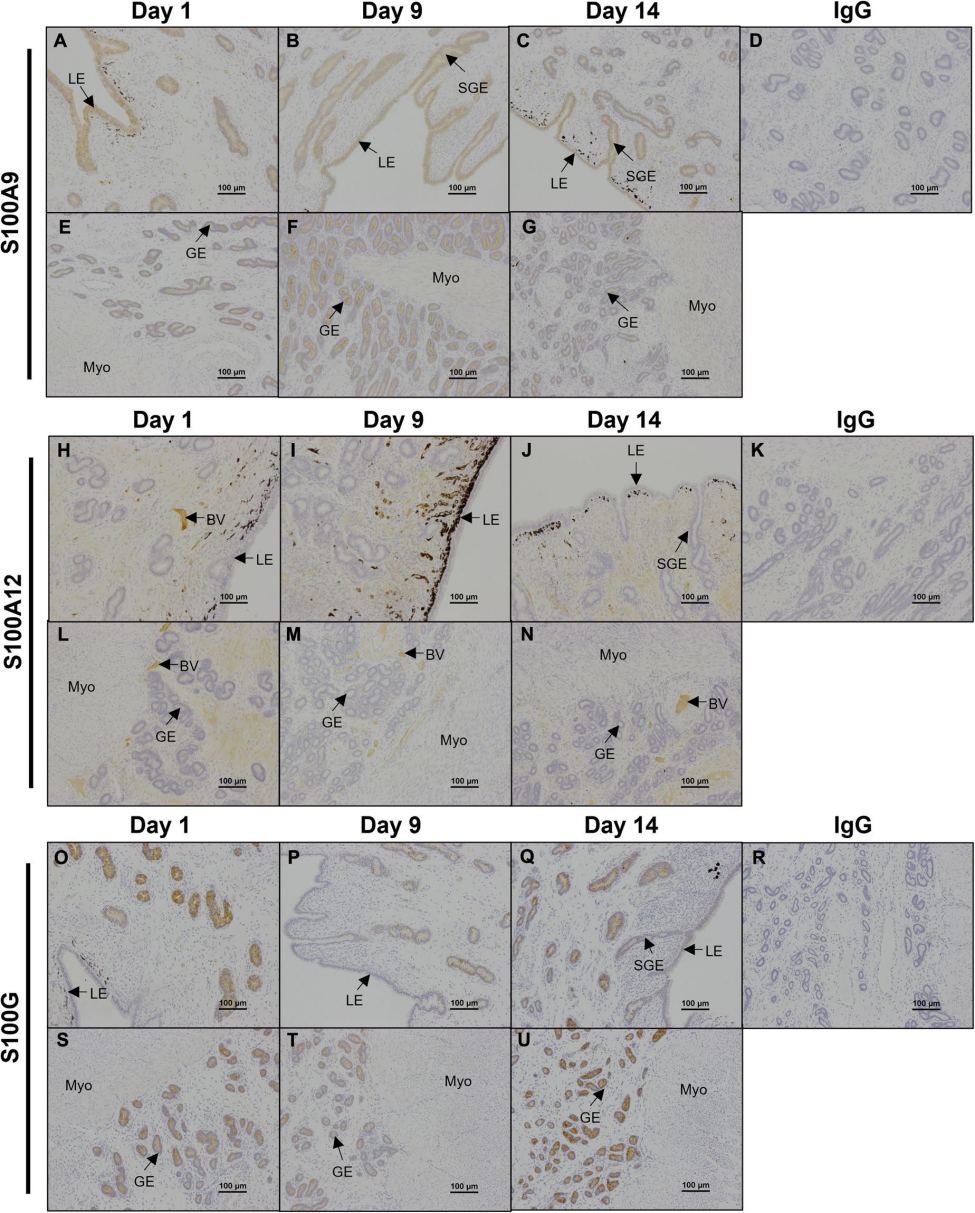
Representative immunolocalization of S100A9, S100A12, and S100G proteins in ovine endometria. Representative images of immunohistochemical localization of the calcium binding proteins S100A9 (**A**-**G**), S100A12 (**H**-**N**), and S100G (**O**-**U**) in ovine endometria on Days 1, 9, and 14 of the estrous cycle. Rabbit IgG (rIgG) controls were included at equivalent protein concentrations to the antibody of interest as a negative control (D, K, R). BV = blood vessels, LE = luminal epithelium, GE = glandular epithelium, sGE = superficial glandular epithelium, Myo = myometrium. Scale bars represent 100 μm

The calcium binding protein S100A9 immunolocalized to uterine LE, SGE, and GE, with weak staining in the myometrium (Fig. 5). In contrast, S100A12 protein localized to the uterine stratum spongiosum stromal cells and blood vessels (Fig. 5). Further, the calcium binding protein S100G localized exclusively to uterine GE (Fig. 5) and was less abundant on Day 9 than on Days 1 and 14 of the estrous cycle.

**Fig. 6:**
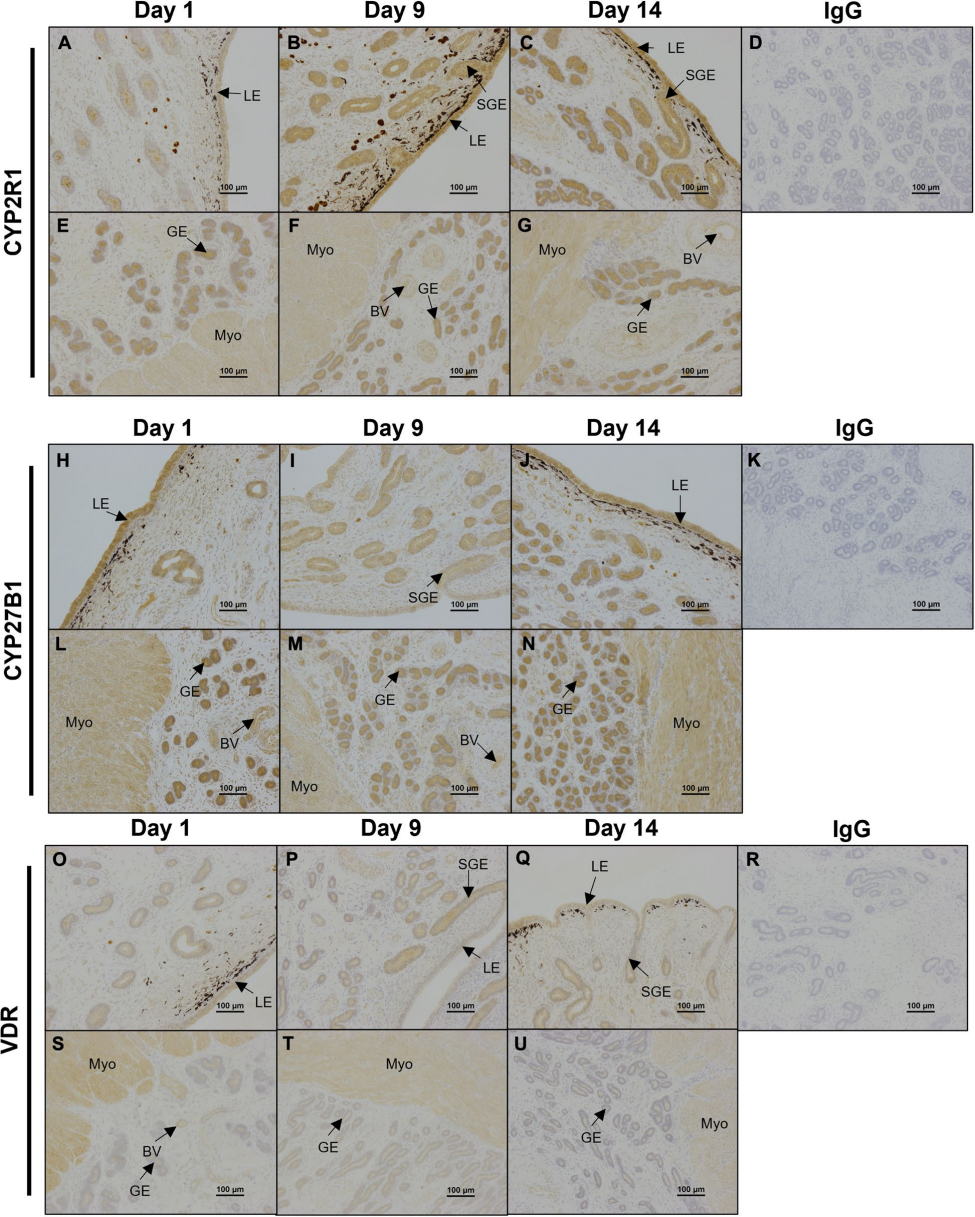
Representative immunolocalization of CYP2R1, CYP27B1, and VDR proteins in ovine endometria. Representative images of immunohistochemical localization of CYP2R1 (**A**-**G**), CYP27B1 (**H**-**N**), and VDR (**O**-**U**) proteins in ovine endometria on Days 1, 9, and 14 of the estrous cycle. Rabbit IgG (rIgG) controls were included at equivalent protein concentrations to the antibody of interest as a negative control (**D**, **K**, **R**). BV = blood vessels, LE = luminal epithelium, GE = glandular epithelium, sGE = superficial glandular epithelium, Myo = myometrium. Scale bars represent 100 μm

The proteins encoding 25-hydroxylase CYP2R1 and 1α-hydroxylase CYP27B1 (enzymes critical for the generation of the active vitamin D hormone 1,25(OH)_2_D_3_) immunolocalized to uterine LE, sGE, GE, myometrium, and blood vessels (Fig. 6). CYP2R1 immunoreactivity appeared stronger on Days 9 and 14 compared to Day 1 of the estrous cycle (Fig. 6). Additionally, vitamin D receptor (VDR) protein immunolocalized to the uterine LE, sGE, GE, Myo, and blood vessels (Fig. 6).

## Discussion

The transport of nutrients, including minerals, across the maternal-conceptus interface is essential for the establishment and maintenance of pregnancy and for the regulation of fetal-placental growth and development [3, 21]. Despite the wide acceptance that phosphate, calcium, and vitamin D are essential for the establishment and maintenance of pregnancy, as well as for the regulation of fetal growth, the hormonal mechanisms regulating transport and metabolism of these fundamental nutrients remains under-investigated and poorly understood. Results of our recent investigations indicate regulatory roles for P4 and IFNT, both alone and synergistically, to affect expression of regulators of calcium, phosphate, and vitamin D signaling, transport, and metabolism in the ovine endometrium in pseudo pregnant ewes [29]. Further, treatment of ewes with exogenous P4 during the pre-implantation period of pregnancy had long-term effects on mineral signaling in both endometria and placentomes in late gestation [33]. However, the effects of changes in E2, ESR1, P4, and PGR on expression of mRNAs and proteins critical for mineral signaling in the endometria of sheep are poorly understood. The results of this study provide compelling evidence that the naturally occurring changes in P4 and E2 and their respective receptors in cells of the uterus across the estrous cycle are consistent with the regulation of phosphate, calcium and vitamin D signaling, transport, and metabolism requirements of the developing fetus.

The abundance of Ca^2+^ in uterine flushings was lower on Day 9 compared to Day 1 of the estrous cycle, which was accompanied by lower immunoreactive S100G protein in uterine GE. As there are high concentrations of E2 in blood and high expression of ESR1 in uterine LE and GE on Day 1, the decrease in S100G protein may suggest regulation of endometrial calcium signaling by E2 acting via ESR1. Postnatally, E2 is considered a calciotropic hormone and an important regulator of bone homeostasis in both males and females that regulates osteoclast function [35]. Furthermore, E2 is a known regulator of calcium transport in the post-natal intestine [36] and kidney [37]. In the rat, fluctuations in concentrations of Ca^2+^ may result from activities of different calcium transporters in uteri that respond differentially to P4 and E2 during the estrous cycle [38, 39]. Additionally, in the pig, E2, the pregnancy recognition signal, increases the release and/or transport of calcium into the uterine lumen during the peri-implantation period of gestation [40].

Perhaps unsurprisingly, the majority of the temporal changes observed in this study may be attributed to P4 and/or PGR signaling. Interestingly, while endometrial expression of *ADAM17*, *FGFR2*, and *PTHrP* mRNAs increased between Days 1 and 9 of the estrous cycle, the expression of these mRNAs decreased between Days 9 and 14 of the estrous cycle perhaps due to down-regulation of PGR expression in uterine LE and sGE. Collectively, these findings suggest that endometrial expression *ADAM17*, *FGFR2*, and *PTHrP* mRNAs is likely regulated by the action of P4 signaling through the PGR for uterine LE and sGE.

Administration of exogenous P4 for the first 8 d of pregnancy is sufficient to advance down-regulation of PGR expression in the uterine LE and sGE by Day 12 of gestation [24]. However, PGR continues to be expressed by the deep uterine GE, stroma, and myometrium [24, 34]. P4 induces the production of growth factors (FGF7, FGF10, and hepatocyte growth factor), collectively termed ‘progestamedins’, by uterine stromal cells to allow P4 to mediate expression of mRNAs and proteins required for functional uterine epithelial cells [41]. In addition to binding phosphatonin FGF23, the progestamedins can also bind to FGFR2 to induce paracrine signaling effects on uterine epithelial cells to stimulate secretion of histotroph and expression of transporters for nutrients [41]. It is important to note that FGF23 not only localizes to uterine epithelial cells, but also localizes to some uterine stromal cells. Ewes treated with exogenous P4 had lower expression of *FGFR2* mRNA than controls on Day 12 of gestation [33]. Further, endometrial expression of *FGFR2* mRNA expression decreases in pregnant ewes between Days 12 and 17 [18].

Parathyroid hormone (PTH) and PTH-related protein (PTHrP) have defining roles in the regulation of extracellular calcium and phosphate metabolism and in controlling skeletal growth and repair [42]. Further, PTHrP has been strongly implicated as a critical regulator of the placental transport of calcium in several mammalian species [3, 21]. In this study, the endometrial expression of *PTHrP* mRNA increased between Days 1 and 9 of the estrous cycle with increasing concentrations of P4, and then decreased between Days 9 and 14 of the estrous cycle when expression of PGR by uterine LE and sGE is downregulated. In cell lines with excessive PTHrP production, steroid hormones negatively regulate PTHrP expression, but ESR1 and PGR are required for the repression of PTHrP [43]. While it is known that PTHrP is a regulator of placental phosphate transport in sheep, little is known regarding expression and hormonal regulation of this molecule or receptor by the ovine endometrium, adding importance to this novel finding.

The endometrial expression of the plasma membrane calcium ATPase *ATP2B4* mRNA increased between Days 9 and 14 and decreased between Days 14 and 1 of the estrous cycle. This suggests a suppressive effect of PGR signaling on the endometrial expression of *ATP2B4* mRNA and, in turn, a potential effect of loss of PGR on the maintenance of calcium homeostasis in the ovine uterus. Plasma membrane Ca^2+^ ATPases (PMCAs) function to maintain intracellular calcium homeostasis and a regulatory role for Ca^2+^ ATPases on calcium transport at the maternal-conceptus interface in humans, rodents, sheep, and pigs has been suggested [44–47]. The expression profile for *PMCA3* and *PMCA4* (also known as *ATP2B3* and *ATP2B4*) mRNAs in ovine endometria and placentomes across gestation suggests a role for these molecules in the regulation of implantation and placental development in sheep [18].

In this study, the apparent increase in immunoreactive KL protein in uterine LE between Days 1 and 9 of the estrous cycle suggests a regulatory role for P4 in KL expression. Further, the expression of *KL* mRNA decreased between Days 9 and 14 of the estrous cycle, suggesting that the actions of P4 on KL expression is regulated by P4 signaling through the PGR in uterine LE and sGE. The phosphatonin FGF23 is considered a significant regulator of phosphate transport through interactions with KL, forming complexes with FGFR that facilitates high affinity binding of FGF19, FGF21, and FGF23 (FGF19 subfamily members). We previously reported that endometrial *KL* mRNA expression is greater on Day 12 than Day 9 of gestation, and that concentrations of P4 and endometrial expression of *KL* mRNA were positively correlated [33]. Therefore, the current findings provide additional evidence that P4 is important for the regulation of the KL signaling pathway.

KL-FGF23 signaling regulates the expression of Type II and III phosphate sodium-dependent transporters (SLC20A1, SLC20A2, and the SLC34 family) postnatally [48, 49]. Like *KL*, endometrial expression of *SLC20A1* mRNA decreased between Days 9 and 14 and increased between Days 14 and 1 of the estrous cycle. In pregnant sheep, endometrial expression of *SLC20A1* mRNA is lower on Day 17 than Day 9 of gestation [18]. In sum, these findings strongly suggest that KL and SLC20A1 are regulated by the actions of PGR signaling in uterine LE and GE and support an important role for P4 in the regulation of phosphate homeostasis in the uterus.

In the present study, endometrial expression of *CYP24* mRNA decreased between Days 9 and 14 of the estrous cycle when down-regulation of PGR expression occurred in the uterine LE and sGE. The activity of vitamin D is regulated by the catabolic activity of CYP24 (24-hydroxylase) which inactivates 1,25(OH)_2_D_3_ via conversion to 1,24,25(OH)_3_D_3_ [50]. Endometrial expression of *CYP24* mRNA is lower on Day 12 than Day 9 of gestation in sheep [33]. Further, administration of exogenous P4 for the first 8 d of pregnancy down-regulated *CYP24* mRNA expression in endometria on Days 9 and 12 of gestation [33]. Thus, these observations provide compelling evidence for a regulatory role of P4 acting via PGR in uterine LE and sGE to influence the expression of *CYP24* mRNA and, in turn, the metabolism of vitamin D by the ovine uterus. We reported high expression of *CYP24* mRNA by the ovine endometrium in early pregnancy which decreased with advancing days of gestation [19]. Postnatally, vitamin D enters the systemic circulation bound to vitamin D binding protein and is transported to the liver, where it is hydroxylated by CYP2R1 to 25-hydroxyvitamin D (25(OH)D) [50]. In the present study, the apparent down-regulation of CYP2R1 protein occurred in endometria between Days 14 and 1 of the estrous cycle. Interestingly, upregulation of CYP2R1 protein immunoreactivity was observed between Days 1 and 9 of the estrous cycle. These temporal changes in CYP2R1 abundance suggests that P4 regulates 25-hydroxylase activity of CYP2R1, thereby increasing the availability of 25(OH)D_3_ within the uterine lumen, presumably for subsequent activation by the abundant 1α-hydroxylase, CYP27B1. In fact, concentrations of P4 in plasma are positively correlated with expression of endometrial *CYP2R1* mRNA and administration exogenous P4 for the first 8 d of pregnancy increased endometrial expression of *CYP2R1* mRNA on Day 125 of gestation [33]. The expression of *VDR* mRNA increased between Days 14 and 1 in concert with increases in E2 in blood and expression of ESR1 by uterine LE and GE in ewes [24]. The endometrial expression of *VDR* mRNA then decreased between Days 1 and 9 of the estrous cycle with the switch from an estrogenic to a progestinized uterine environment. Collectively, these findings suggest a localized negative feedback mechanism to regulate the abundance of vitamin D in the ovine uterus that is regulated by P4 via PGR to play an important role in the local metabolism of vitamin D in the uterus to meet nutritional demands of the conceptus.

In addition, concentrations of 25(OH)D in plasma increased between Day 1 and 14 of the estrous cycle, also suggesting a role for P4/PGR signaling in regulating systemic vitamin D status. Similar findings have been reported for cyclic ewes in which treatment with RU486 (mifepristone, PGR antagonist) and P4 decreased 25(OH)D compared to values for ewes which were treated with only P4 [29]. It is interesting to speculate that P4 also alters the activity of the hydroxylases in the maternal liver or kidney that are critical for the generation of 25(OH)D_3_ and 1,25(OH)_2_D_3_, leading to systemic alterations in maternal vitamin D status.

## Conclusion

The results of this study provide compelling evidence that the naturally occurring changes in P4 and E2, along with PGR and ESR1, across the estrous cycle are important for the regulation of phosphate, calcium and vitamin D signaling, transport, and metabolism. Given the exceptionally high rate of embryonic mortality in eutherian mammals and the importance of steroid hormones as regulators of nutrient transport, this study provides an important platform for further mechanistic research to improve the understanding of mechanisms governing the establishment of pregnancy, conceptus growth, and successful outcomes of pregnancy in the livestock species. Considering the conserved nature of the functions of E2 and P4 across species, and the mechanisms regulating mineral homeostasis postnatally across species, these findings may also have important implications for pregnancy outcomes in humans.

## Supplementary Information

**Additional file 1:** **Table S1.** Primer sequences.

**Additional file 2:** **Table S2.** Antibodies used.

## Data Availability

The datasets used and/or analyzed during the current study are available from the corresponding author on reasonable request.

## Abbreviations

cDNA: Complementary DNA
EDTA: Ethylenediaminetetraacetic acid
ELISA: Enzyme-linked immunoassay
E2: Estradiol
ESR1: Estrogen receptor
GE: Glandular epithelium
IFNT: Interferon tau
LE: Luminal epithelium
P4: Progesterone
PGR: Progesterone receptor
qPCR: Quantitative polymerase chain reaction
RU486: Mifepristone
sGE: Superficial glandular epithelium
