# Supplementary material for: Phosphate, calcium, and vitamin D signaling, transport, and metabolism in the endometria of cyclic ewes
Source: J Anim Sci Biotechnol. 2023 Jan 12;14:13. doi: 10.1186/s40104-022-00803-2 (PMC9835233; doi:10.1186/s40104-022-00803-2)
Supplement: Supplementary file 1 — Additional file 1: Table S1. Primer sequences. [file 40104_2022_803_MOESM1_ESM.docx]

**Table S1** Primer sequences

| **Gene symbol** | **Gene name** | **Accession number** | **Primer sequence (5’🡪 3’)** | | **Tm, ^o^C** | **Amplicon size** |
| --- | --- | --- | --- | --- | --- | --- |
| *ADAM10* | A Disintegrin and metalloproteinase domain-containing protein 10 | XM_004010565.4 | Fwd | TGTGCCAGTTCTGATGGCAA | 60 | 103 |
|  |  |  | Rev | TCCACTGCACAGACCCTGTA |  |  |
| *ADAM17* | A Disintegrin and metalloproteinase domain-containing protein 17 | XM_004005676.4 | Fwd | ATGGCAAGTGTGAGAAGCGA | 60 | 117 |
|  |  |  | Rev | GGACGGAACCGACGATGTTA |  |  |
| *ATP2B4* | Plasma membrane calcium-transporting ATPase 4 | XM_027976182.1 | Fwd | TTGACAGCGGAAGGAGAGC | 60 | 117 |
|  |  |  | Rev | CGTGGATCTTGCGGGAGTT |  |  |
| *B2M* | Beta-2-microglobulin | XM_012180604.2 | Fwd | CATCTTAGCGGTGTGGAGGG | 60 | 72 |
|  |  |  | Rev | TTGTGTGCAAAACACCCTGAC |  |  |
| *CYP2R1* | Cytochrome P450 Family 2 Subfamily R Member 1 | XM_004016131.4 | Fwd | GAAGCCCAGGTTAGGCATGA | 60 | 100 |
|  |  |  | Rev | GGCAAACAGCTTGTTCCACA |  |  |
| *CYP24* | 25-hydroxyvitamin D3-24-hydroxylase | XM_027976458.1 | Fwd | AACGGTGGCTTCAGGACAAG | 60 | 82 |
|  |  |  | Rev | CGACCCACGCACATTCTTTT |  |  |
| ***FGF23*** | **Fibroblast growth factor 23** | **XM_027967989.1** | **Fwd** | **ATTCCGGGTTTTCCCATACACA** | **60** | **121** |
|  |  |  | **Rev** | **AACAACAAGCTAGAGTGACCAGA** |  |  |
| *FGFR1* | Fibroblast growth factor receptor 1 | XM_027962628.1 | Fwd | ACAAGATGAAGAGCGGCACA | 60 | 107 |
|  |  |  | Rev | GTCGGCTGACACTGTTACCT |  |  |
| *FGFR2* | Fibroblast growth factor receptor 2 | XM_027960356.1 | Fwd | CCTGCGGAGACAGGTAACAG | 60 | 137 |
|  |  |  | Rev | GCAGCTCATACTCGGAGACC |  |  |
|  |  |  | Rev | TTGGAGGCTCGACAGAGGTA |  |  |
| *KL* | Klotho | XM_004012279.4 | Fwd | GCCTGCACATAGGGGACTTT | 60 | 120 |
|  |  |  | Rev | CTCCAGCCACTGCGCTATAA |  |  |
| *PTHrP* | Parathyroid hormone-related peptide | XM_004006756.3 | Fwd | CCTAGTTCGCAAAGAAGCTGAC | 60 | 71 |
|  |  |  | Rev | GCAGGGCTAACTCCTTCCTA |  |  |
| *SLC20A1* | Solute carrier family 20 member 1 | XM_004005910.4 | Fwd | CTGCTTCCAGGTTCTGTCGT | 60 | 92 |
|  |  |  | Rev | CGGACGCACGCACCTTTTAT |  |  |
| *S100A9* | S100 calcium-binding protein A9 | XM_012181723.3 | Fwd | CTATACGGCTAAGGGACCCG | 60 | 72 |
|  |  |  | Rev | TTGGCAGCTCTTTTTGCACC |  |  |
| *S100G* | S100 calcium-binding protein G | XM_004021937.3 | Fwd | GCAGCCAAAGAAGGTGATCC | 60 | 73 |
|  |  |  | Rev | TGGGGAATTCCGTCTGAAGC |  |  |
|  |  |  | Rev | TGACGTTCACCTTCTTGGCA |  |  |
|  |  |  | Rev | ACGGTTTTCCCTTTTTAGCAGC |  |  |
| *TRPV6* | Transient Receptor Potential Cation Channel Subfamily V Member 6 | XM_004008129.4 | Fwd | TTTGCGCGAGGATTCCAGAT | 60 | 90 |
|  |  |  | Rev | CATCAGCCAGCAGAACCTCA |  |  |
| *VDR* | Vitamin D Receptor | XM_027967403.1 | Fwd | AGTTCGCAAGGATGAGGACG | 60 | 84 |
|  |  |  | Rev | CAGGAGGACGACGAGTTTCC |  |  |
| *YWHAZ* | Tyrosine 3-monooxygenase/tryptophan 5-monooxygenase activation protein zeta | NM_001267887.1 | Fwd | GACTGGGTCTGGCCCTTAAC | 60 | 72 |
|  |  |  | Rev | GAGAGCAGGCTTTCTCAGGG |  |  |

Bold indicates that preamplification was performed for 15 cycles prior to performing the qPCR
