# Supplementary material for: Phosphate, calcium, and vitamin D signaling, transport, and metabolism in the endometria of cyclic ewes
Source: J Anim Sci Biotechnol. 2023 Jan 12;14:13. doi: 10.1186/s40104-022-00803-2 (PMC9835233; doi:10.1186/s40104-022-00803-2)
Supplement: Supplementary file 2 — Additional file 2: Table S2. Antibodies used. [file 40104_2022_803_MOESM2_ESM.docx]

**Supplementary Table 2: Antibodies Used**

| **Antibody** | | **Company** | **Product Number** | **Research Resource Identifier** | **Isotype** | **Clonality** | **Species Raised In** | **Antigen Retrieval** | **Concentration, mg/mL** |
| --- | --- | --- | --- | --- | --- | --- | --- | --- | --- |
| Primary | ADAM17 | Bioss | bs-12447 | Not available | IgG | Polyclonal | Rabbit | Citrate | 0.0014 |
| Primary | CYP2R1 | Bioss | bs-3900 | AB_10857030 | IgG | Polyclonal | Rabbit | Tris | 0.006 |
| Primary | CYP27B1 | Bioss | bs-14146 | Not available | IgG | Polyclonal | Rabbit | Tris | 0.006 |
| Primary | FGF23 | Bioss | bs-5768R | AB_11051406 | IgG | Polyclonal | Rabbit | Citrate | 0.003 |
| Primary | KL | Bioss | bs-2925R | AB_11078665 | IgG | Polyclonal | Rabbit | Citrate | 0.005 |
| Primary | S100A9 | Abcam | ab92507 | AB_10562628 | IgG | Monoclonal | Rabbit | Citrate | 0.002 |
| Primary | S100A12 | Proteintech | 16630 | AB_2878290 | IgG | Polyclonal | Rabbit | Tris | 0.002 |
| Primary | S100G | Invitrogen | PA5-63195 | AB_2646898 | IgG | Polyclonal | Rabbit | Citrate | 0.001 |
| Primary | VDR | Bioss | bs-2987R | AB_11083851 | IgG | Polyclonal | Rabbit | Citrate | 0.004 |
| Secondary | Biotinylated anti-rabbit IgG | Vector Laboratories | PK-6200 | AB_2336826 |  | Not Provided | Horse |  | 0.005 |
